# A vast dataset for Kurdish handwritten digits and isolated characters recognition

**DOI:** 10.1016/j.dib.2023.109014

**Published:** 2023-03-02

**Authors:** Peshraw Ahmed Abdalla, Abdalbasit Mohammed Qadir, Mohammed Y. Shakor, Ari M. Saeed, Abdalla Taha Jabar, Ali Abdalla Salam, Hedi Hamid Hama Amin

**Affiliations:** a Department of Computer Science, College of Science, University of Halabja, Halabja, Iraq; b Department of Computer Science, College of Science and Technology, University of Human Development, Sulaimaniyah, Iraq; c Department of English, College of Education, University of Garmian, Kalar, Iraq

**Keywords:** Central Kurdish, Characters and digits images, Kurdish optical character and digit recognition, Word segmentation

## Abstract

This article presents two massive datasets for central Kurdish handwriting digits and isolated characters named *K-ZHMARA* and *K-PIT*. The first dataset, named *K-ZHMARA* dataset, contains 70,000 images of Kurdish digits, 7000 images for each digit, and a printed A4 paper with a grid of 10 × 10 is used for data collection. Apart from digits, the *K-PIT* dataset includes 245,000 images of all Kurdish characters, 7000 images for each character; data was collected via a printed A4 paper with a grid of 12 × 10 for this dataset. Moreover, both datasets include 315,000 images. Python programming has been used to scan each piece of paper, segment, crop, resize, binarize, and invert the images via edge detection and image processing techniques.

**Specifications Table**

**Table:** 

Subject	Pattern Recognition, Computer Vision, and Deep Learning
Specific subject area	A vast dataset for Kurdish digits and isolated characters recognition
Type of data	Image
How data were acquired	The collected handwriting images were captured using a scanner and then segmented, cropped, resized, binarized, inversed, and annotated.
Data format	Raw data Segmented data Annotations Jpg format
Description of data collection	Each handwritten digit and character were written on an empty printed grid paper to facilitate the segmentation process. Writers were advised to write the digits and characters in the right boxes according to its label. Data collection forms were collected from more than 1500 participants. The handwritten characters and digits are segmented using bounding boxes. Each of the bounding boxes contains the characters that are written.
Data source location	Halabja, Kurdistan Region, Iraq
Data accessibility	A vast dataset for Kurdish digits and isolated characters recognition [1]. Data identification number: 10.17632/zb66pp7vjh.1 Direct URL to data: https://dx.doi.org/10.17632/zb66pp7vjh.1

## Value of the Data

• In the study of pattern recognition and image processing, handwriting recognition is regarded as an exciting and motivating problem. It can be used in a variety of ways, such as applications to learn the characters and digits for children, reading assistance for the blind, computerized reading, processing for paper documents, and turning any handwritten material into structural text.
• The datasets can be used for handwriting optical character/digit recognition and identification using machine learning and deep learning models.
• Both datasets are ready to implement since they are pre-processed (including suspending excess lighting and noises, segmentation, cropping, resizing, binarization image, and inverse images) for each character and digit.
• Deep learning and machine learning researchers are interested in central Kurdish or other languages with similar scripts, such as Persian, Arabic, and Urdu.
• The datasets can be used as a standard for usability and quality in subsequent works because they were collected in a precise way, and it is vast data that can achieve higher accuracy in designed models.

## 1 Objective

OCR aims to modify or convert any type of text or text-containing document, including handwritten, printed, or scanned text images, into a digital format that may be edited and used for more in-depth processing. OCR allows a machine to recognize text in such materials automatically. A few significant obstacles must be identified and overcome to automate successfully, for instance, the existence of a huge and reliable dataset.

There has not been much research done on automatically recognizing Kurdish handwritten characters and digits since machine and deep learning models need huge datasets to achieve high accuracy; the aim of this work is to prepare two huge datasets for the Kurdish language named K-PIT (for Kurdish characters) and K-ZHMARA (for Kurdish digits), these datasets can be used to build a model for handwriting optical character/digit recognition and identification via deep learning and machine learning approaches.

## 2 Data Description

Kurdish language dialects are used across four main nation-states in the Middle East [2], and only one dialect, Sorani, has official status in one of these nation-states. The majority of Kurdish-speaking regions are located in Turkey, Iraq, Iran, and Syria. More than 40 million people speak Kurdish as a whole, according to estimates [3,4]. One of the two main dialects of Kurdish, known as Central Kurdish (Sorani), is spoken by an estimated 9 to 10 million people [5]. It is mostly written with a 35-character modified Arabic/Persian alphabet without characters that have recently been replaced, such as (ك), which is no longer used by the Kurdish language and has been replaced with (ک) [6,7]. A large database of isolated handwritten Central Kurdish digit and character images has been developed in this effort, totaling 315,000 images, with 7000 images of each handwritten by more than 1500 native individuals. Table 1 shows the number of images and the percentage of each character in the K-PIT database. The Quantity and Proportion of Digits Obtained for the K-ZHMARA Dataset are shown in Table 2. Central Kurdish uses modified Arabic/Persian (Farsi) characters for writing, and there are numerous expansive databases of Persian and Arabic handwriting characters for recognition of offline characters; some databases even assert that their database can be used to recognize other languages that use the Arabic scripts, for instance, Kurdish [8], [9], [10]. Nevertheless, there are three primary issues. The first is that it does not include all of the Kurdish letters, such as V(ڤ), L (ڵ), J(ژ), R(ڕ), and O (ۆ). The Kurdish language has an inconsistent quantity and percentage of characters, which is the second issue. The third problem is all the datasets worked with the characters only and ignored the digits.

**Table 1:** Quantity and proportion of characters obtained for the K-PIT dataset.

NO.	Kurdish machine alphabetic	Kurdish handwritten alphabetic	Number of images	Percentage
1	ئـ		7000	2.85%
2	ا		7000	2.85%
3	ب		7000	2.85%
4	پ		7000	2.85%
5	ت		7000	2.85%
6	ج		7000	2.85%
7	چ		7000	2.85%
8	ح		7000	2.85%
9	خ		7000	2.85%
10	د		7000	2.85%
11	ر		7000	2.85%
12	ڕ		7000	2.85%
13	ز		7000	2.85%
14	ژ		7000	2.85%
15	س		7000	2.85%
16	ش		7000	2.85%
17	ع		7000	2.85%
18	غ		7000	2.85%
19	ف		7000	2.85%
20	ق		7000	2.85%
21	ڤ		7000	2.85%
22	ک		7000	2.85%
23	گ		7000	2.85%
24	ل		7000	2.85%
25	ڵ		7000	2.85%
26	م		7000	2.85%
27	ن		7000	2.85%
28	هـ		7000	2.85%
29	ه		7000	2.85%
30	و		7000	2.85%
31	وو		7000	2.85%
32	ۆ		7000	2.85%
33	ى		7000	2.85%
34	ێ		7000	2.85%
35	ص		7000	2.85%
			245,000	100%

**Table 2:** Quantity and proportion of digits obtained for the K-ZHMARA dataset.

NO.	Kurdish machine digits	Kurdish handwritten digits	Number of images	Percentage
1	٠		7000	10%
2	١		7000	10%
3	٢		7000	10%
4	٣		7000	10%
5	٤		7000	10%
6	٥		7000	10%
7	٦		7000	10%
8	٧		7000	10%
9	٨		7000	10%
10	٩		7000	10%
			70,000	100%

## 3 Experimental Design, Materials and Methods

The data collection methodology for preparing a handwritten image dataset includes several phases, such as gathering handwritten data from participants via designed forms which are labelled to indicate the type and position of the character/digit. The second step is a scanner device that scans the collected data in forms. Next, the scanned forms are processed and segmented in order to extract each digit or character as a separate image. Then some pre-processing techniques are applied to the images in order to achieve higher accuracy percentage when the dataset is used for machine learning models such as binarization and inverting the images. The last step is labeling; each similar digit or character is stored in a specific folder for both test and train directories with unique IDs.

### 3.1 Data Collection

**Fig. 1:**
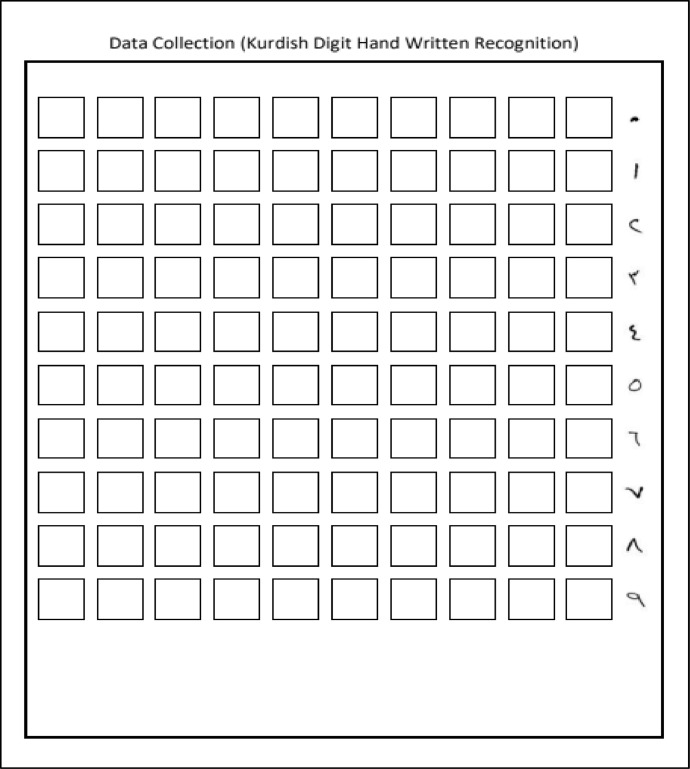
An empty data form for Kurdish handwritten digits.

The first step in creating a database is often locating an appropriate data source. Here, gathering examples of handwritten Kurdish numbers and characters from several different writers is the main objective. This task can be achieved by designing several suitable forms for Kurdish digits and characters. Fig. 1 demonstrates a form designed to collect Kurdish digits for the K-ZHMARA dataset, and it contains 100 empty boxes; each line is labeled with a specific number, and the participants have to fill the forms according to the labels.

**Fig. 2:**
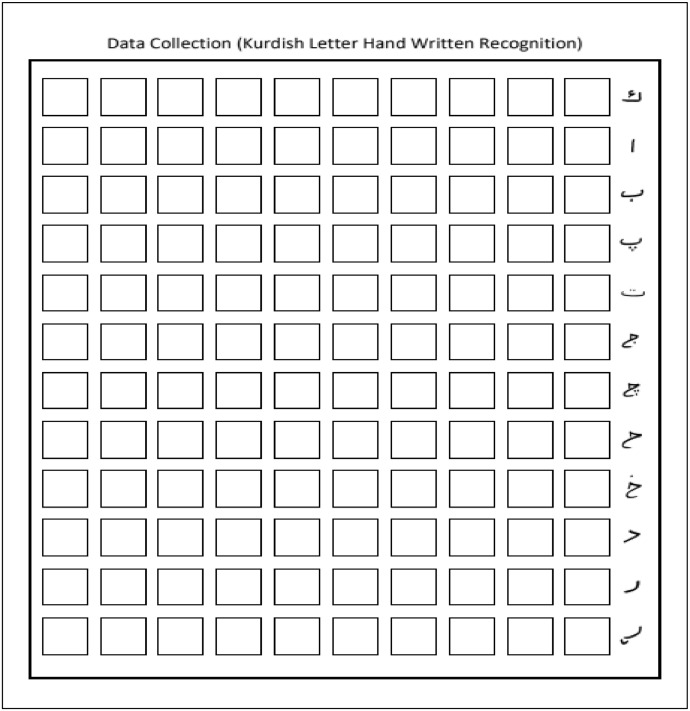
Kurdish handwritten letters empty form.

Each digit is to be written ten times by the writers in each empty line. The number of individuals who participated in building the K-ZHMARA dataset was approximately 700. Similarly, Fig. 2 is another example designed to collect the Kurdish characters for the K-PIT dataset, and it contains 120 empty boxes; each line is labeled with a specific character. Since the Kurdish language (Sorani) has 35 characters without characters that have recently been replaced, we designed three forms, two forms with 12 characters and the last form with 11 characters. Each participant was asked to fill out all three forms. Each character is to be written ten times by the writers in each empty line. The number of individuals who participated in building the K-ZHMARA and K-PIT dataset is more than 1500. The total number of the forms used to collect Kurdish digits was 700 and for the Kurdish characters was 2100, meaning that there were 2400 forms filled out by the volunteers who built the datasets. Several places were selected to fill the forms, students from 5 different colleges of the University of Halabja, the university students who stay in the dormitory of the University of Halabja, and several primary and preparatory schools in Halabja governate; as a result, each character have 700 distinct images.

### 3.2 Processing of Forms

**Fig. 3:**
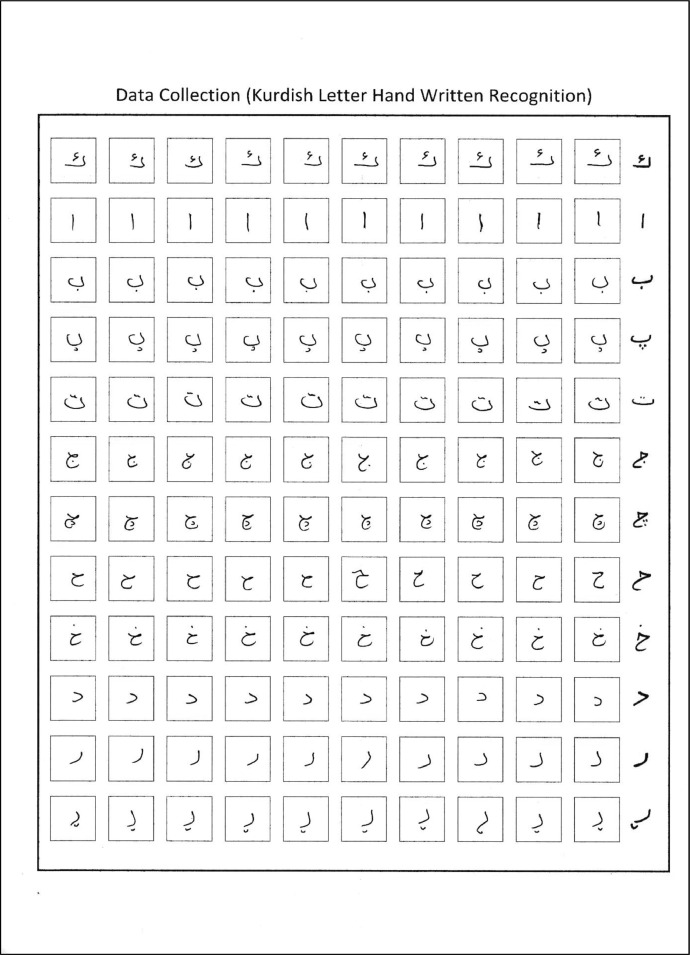
Scanned page example for the K-PIT dataset.

The forms were filled out by the participants and scanned via a scanner. The scanner may produce files in the following formats: pdf, jpeg, png, or tiff. The scanner uses 75 to 600 dpi to scan documents. The png format was chosen for the initial scan of the forms since JPEGs contain less data than PNGs. Due to the forms being white, all forms were filled out using a black or dark blue pen. Fig. 3 displays a sample of a scanned page.

### 3.3 Image Pre-Processing

**Fig. 4:**
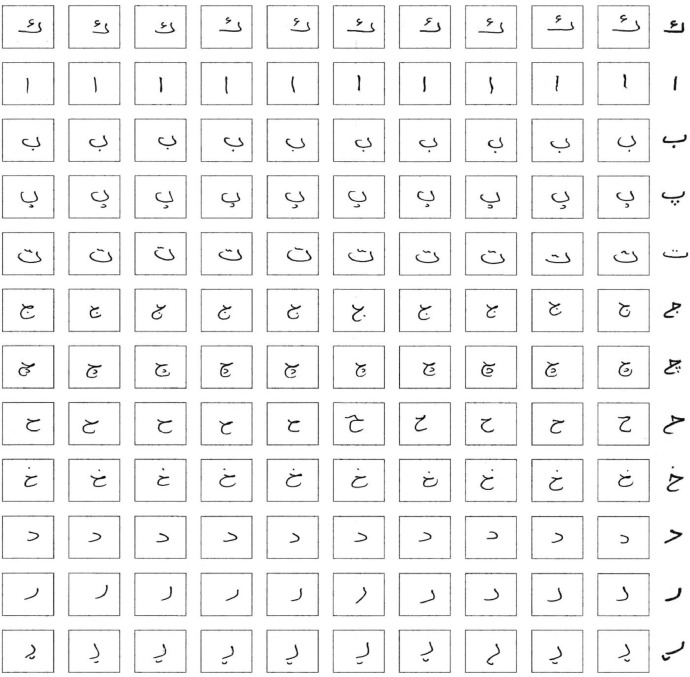
Scanned page after applying the 4-point detection algorithm.

Pre-processing stage is a crucial step in every recognition system. It is used to enhance the quality of pictures. First, the big square border has been detected using Python programming language and 4 point detection algorithm to correct the position and angle of the forms since some forms at the time of scanning may be scanned with an incorrect angle. Fig. 4 Demonstrates a form after applying the 4-point detection algorithm.

### 3.4 Image Segmentation and Cropping

**Fig. 5:**
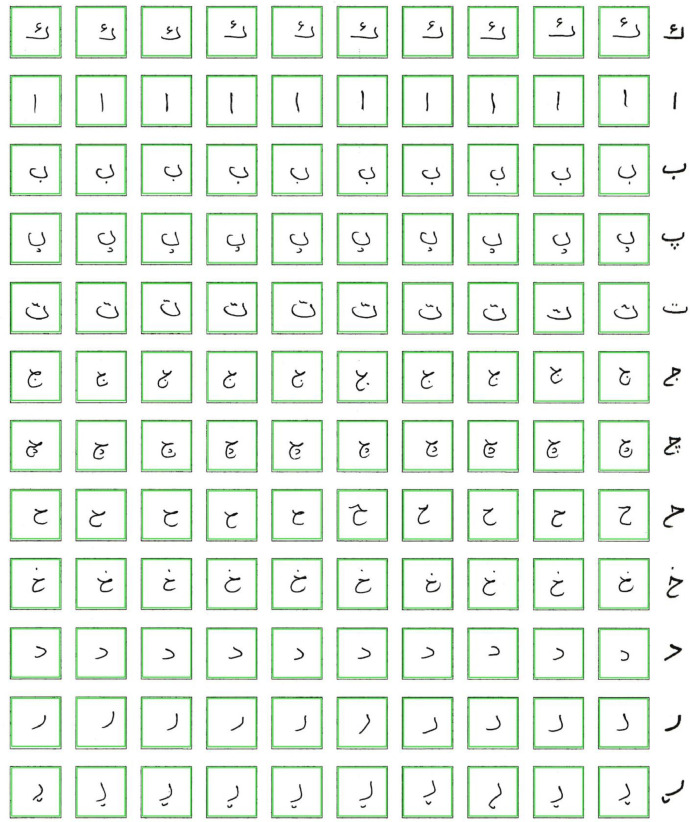
A filled form with all squares around the characters detected by edge detection methods.

Each form page was subjected to the cropping procedure after the pre-processing stage in order to crop each letter block. Each square around the letters and digits has been detected one by one from the first row (left to right). Once all the squares from the first row are detected, and then the program detects the first square from the second row, this procedure will continue until detecting all the squares; this process was done via Python programming and edge detection algorithms, as demonstrated in Fig. 5. The template had different resolutions, and it has 4 different forms, 1 form to collect the digits, which is divided into 10 rows and 10 columns, and 3 forms to collect the characters, 2 of them divided into 12 rows and 10 columns, and the last one divided into 11 rows and 10 columns because the number of Kurdish characters is 35. 100 distinct single-digit picture were created from a page with (146 × 146) pixels when the K ZHMARA dataset template was saved.

**Fig. 6:**
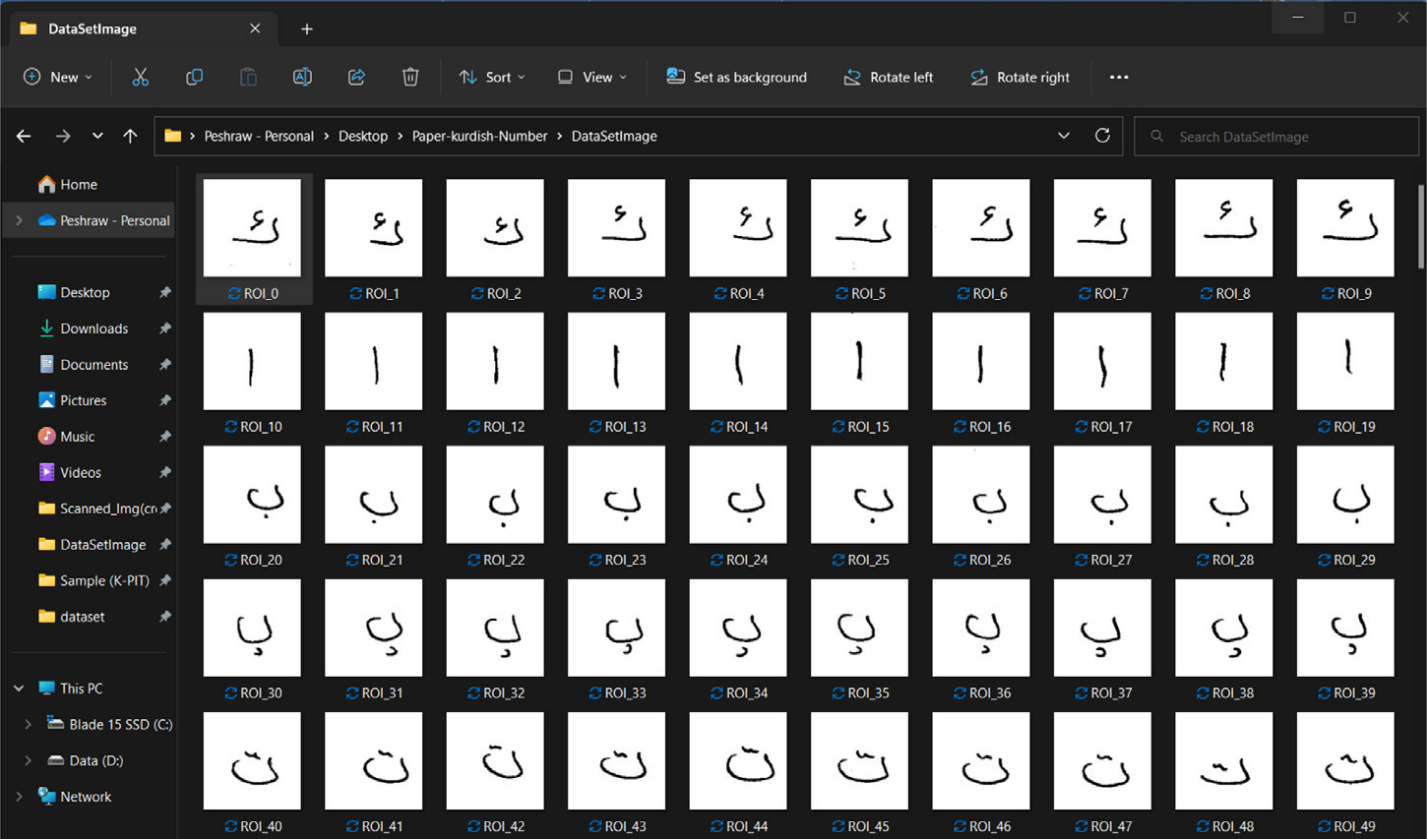
The characters after cropping.

**Fig. 7:**
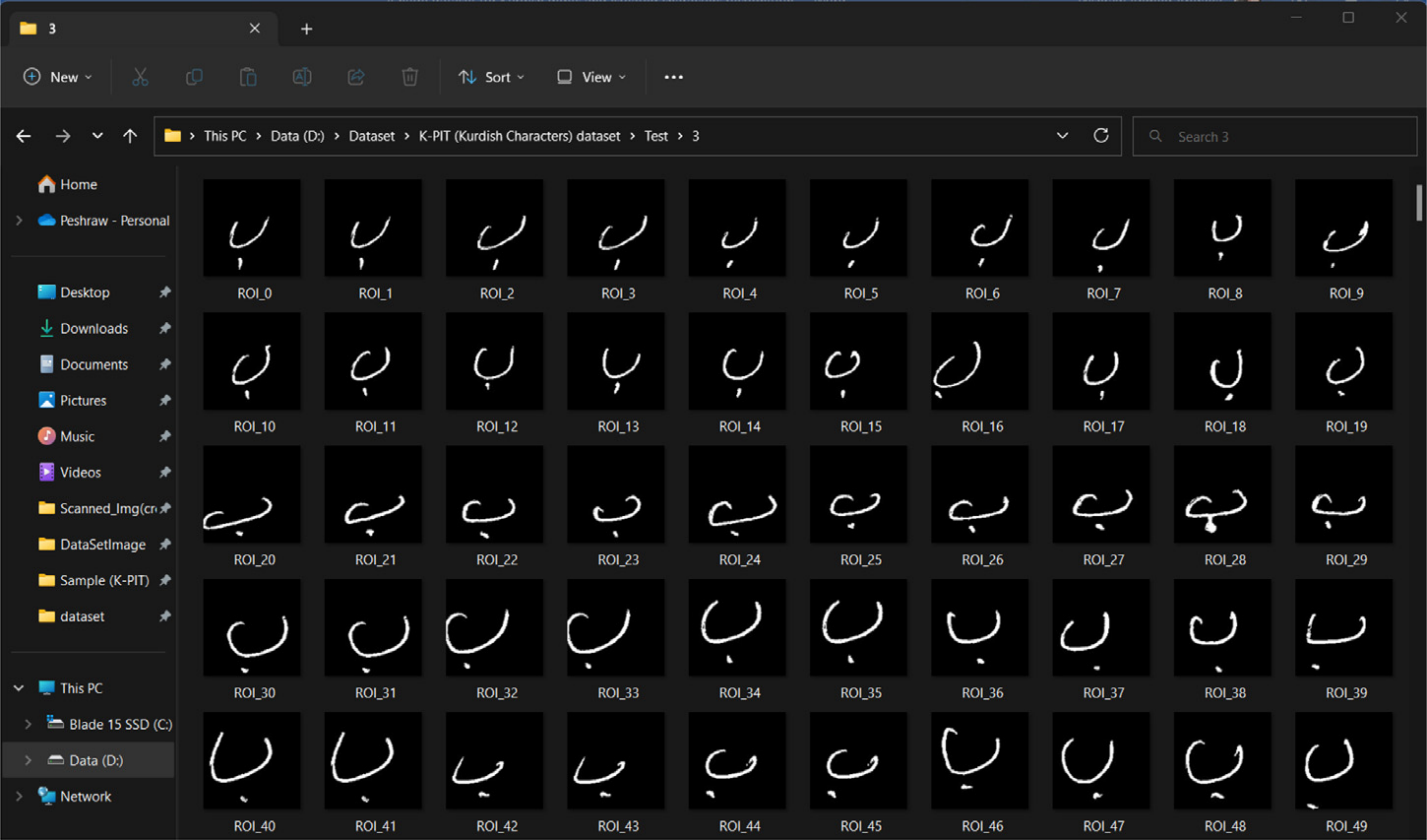
Result of inversing process.

When the templates of the K-PIT dataset were saved, totally generated 350 separate images, 10 images of every single character (the first form, which was labeled with 12 characters, generated 120 images; similarly, the second form generated 120 images, but the last form which labeled with 11 characters generate 110 images) from the page with the (146  ×  146) pixels, the images after cropping, resizing, and the saving process is shown in Fig. 6. Then each line with a specific character/digit is saved in separate folders as a final step and achieves better results with the machine and deep learning models, and all the cropped images are inversed and binarized, as illustrated in Fig. 7.

Each letter or digit was cropped as a separate image during cropping and then saved in a separate folder with its own ID. The images have the same size within the entire dataset. Due to each digit and character being written 10 times by 700 writers, who each wrote once, there were 7000 images produced for each digit and character.

### 3.5 Labeling and Organizing

Labeling is the last step after pre-processing the dataset. Each image is labeled with an ID number, as shown in Table 3 and Table 4; the number of the folder in each dataset represents a single digit or character. For example, folder number 02 in the K-PIT dataset is the id of the letter, which in this case is Alef (ا), and folder number 03 in the K-ZHMARA dataset is the id of the digit, which in this case is three (٣). Each digit and character was stored in a folder with its ID as the name of that folder, with each folder containing 6000 images of that letter/digit for the training and 1000 images for the testing.

**Table 3:** Letter IDs.

ID	Letter	ID	Letter
1	ئـ	19	ف
2	ا	20	ق
3	ب	21	ڤ
4	پ	22	ک
5	ت	23	گ
6	ج	24	ل
7	چ	25	ڵ
8	ح	26	م
9	خ	27	ن
10	د	28	هـ
11	ر	29	ە
12	ڕ	30	و
13	ز	31	وو
14	ژ	32	ۆ
15	س	33	ی
16	ش	34	ێ
17	ع	35	ص
18	غ

**Table 4:** Digit IDs.

ID	Digit	ID	Digit
0	٠	5	٥
1	١	6	٦
2	٢	7	٧
3	٣	8	٨
4	٤	9	٩

## Ethics Statement

After submitting this project to the Institutional Review Board (IRB) of “The University of Halabja.” They accepted this project by a reference protocol (1/8/205) on 1/10/2022.

With the approval of the people who had taken part in the writing, all the handwriting was collected.

## CRediT authorship contribution statement

**Peshraw Ahmed Abdalla:** Supervision, Data curation, Conceptualization, Software, Methodology, Visualization, Project administration, Funding acquisition, Writing – original draft, Writing – review & editing. **Abdalbasit Mohammed Qadir:** Validation, Writing – review & editing. **Mohammed Y. Shakor:** Writing – review & editing. **Ari M. Saeed:** Validation, Writing – review & editing. **Abdalla Taha Jabar:** Software, Data curation, Investigation, Resources. **Ali Abdalla Salam:** Software, Data curation, Investigation, Resources. **Hedi Hamid Hama Amin:** Data curation, Investigation.

## Declaration of Competing Interest

The authors declare that they have no known competing financial interests or personal relationships which have, or could be perceived to have, influenced the work reported in this article.

## Data Availability

A Vast Dataset for Kurdish Digits and Isolated Characters Recognition (Original data) (Mendeley Data).
